# Physicochemical and surface properties of acrylic intraocular lenses and their clinical significance

**DOI:** 10.1007/s40005-017-0323-y

**Published:** 2017-03-27

**Authors:** Gyeong Bok Jung, Kyung-Hyun Jin, Hun-Kuk Park

**Affiliations:** 1 Department of Physics Education, Chosun University, 309 Pilmun-daero, Dong-gu Gwangju, 61452 Republic of Korea; 2 Department of Ophthalmology, College of Medicine, Kyung Hee University, Seoul, 02447 Republic of Korea; 3 Department of Biomedical Engineering & Healthcare Industry Research Institute, College of Medicine, Kyung Hee University, 26 Kyungheedae-ro, Dongdaemun-gu Seoul, 02447 Republic of Korea

**Keywords:** Intraocular lens (IOLs), Cataract surgery, Atomic force microscopy (AFM), Raman spectroscopy, Surface characteristics, Biocompatibility

## Abstract

To analyze and compare several commercially available acrylic intraocular lenses (IOLs) with particular regard to their clinical significance, we examined the physicochemical and surface properties of four currently available acrylic IOLs using static water contact angle, atomic force microscopy (AFM), Raman spectroscopy, and differential scanning calorimetry (DSC) measurements. The hydrophobic acrylic IOLs, ZA9003, and MA60BM, had contact angles ranging from 77.9° ± 0.65° to 84.4° ± 0.09°. The contact angles in the hydrophilic acrylic (970C) and heparin-surface-modified (HSM) hydrophilic acrylic IOLs (BioVue) were 61.8° ± 0.45° and 69.7° ± 0.76°, respectively. The roughness of the IOL optic surface differed depending on the type of IOL (*p* < 0.001). The surface roughness of BioVue had the lowest value: 5.87 ± 1.26 nm. This suggests that the BioVue IOL may lead to reduced cellular adhesion compared to the unmodified IOLs. All IOLs including those composed of acrylic optic materials from different manufacturers showed distinct Raman spectra peaks. The glass transition temperatures (T_g_) for the hydrophobic acrylic IOLs were between 12.5 and 13.8 °C. These results suggest that the intraoperative and postoperative behavior of an IOL can be predicted. This information is also expected to contribute greatly to the industrial production of reliable biocompatible IOLs.

## Introduction

**Fig. 1:**
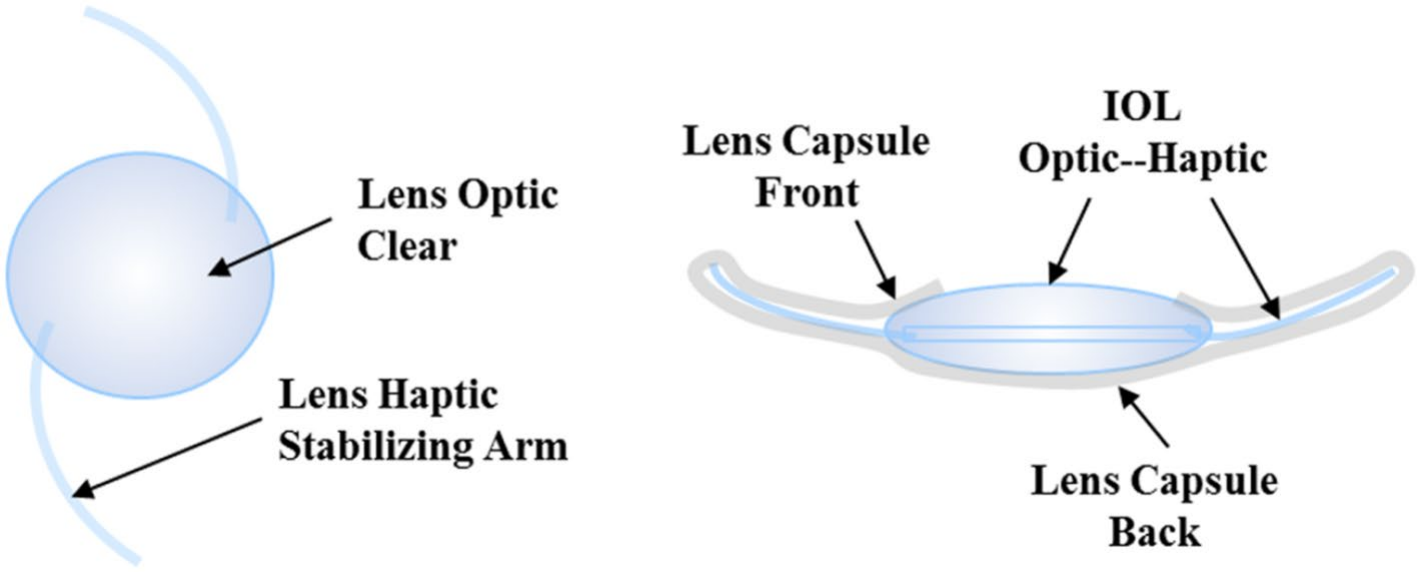
Diagram of intraocular lens (IOL) structure and side view of the IOL within the lens capsule

A cataract is a clouding of the lens in the eye and normally occurs as part of the aging process. When a cataract develops, light is unable to pass directly through lens to the retina, resulting in blurred vision. During cataract surgery, the natural lens of eye is replaced by an artificial lens [intraocular lens (IOL)]. This IOL is placed in the lens capsule (Fig. 1), where it remains after cataract surgery. Since foldable IOL implantation was first used to replace extracted cataractous lenses, there have been many improvements in cataract surgery. However, some patients may have difficulties with vision again a few months to a few years after cataract surgery. This is not a re-growth of cataract; it is because of the thickening of the back of the lens capsule. This is called posterior lens capsule opacification (PCO), which is also referred to as “secondary cataract” or “after cataract.” PCO results from the growth and abnormal proliferation of lens epithelial cells (LECs) on the posterior capsule. This causes cloudy vision. If this happens, laser treatment may be needed to make vision clear again. Therefore, posterior capsule opacification (PCO) is an issue of concern for most cataract surgeons (Apple et al. 2001; Findl et al. 2005; Hollick et al. 1999; Kohnen et al. 2008).

IOLs usually consist of small optics with side structures (haptics) to hold the lens in place within the capsular bag inside the eye. The IOL is inserted into the capsule with the arms (haptics) keeping the lens positioned in the center. Figure 1 shows a diagram of IOL structure and a side view of the IOL within the lens capsule. The optic design and development of more biocompatible IOLs has attracted considerable attention with the aim of preventing PCO (Nishi et al. 2004; Prinz et al. 2011; Saika 2004; Vasavada et al. 2011). The IOL materials most commonly used are polymethyl methacrylate (PMMA), silicone, acrylic, and hydrogel (Barrett 1994; Seward 1997). Cellular proliferation on an IOL surface has been reported as a good indicator of biocompatibility of the lens material. Tanaka et al. (2005) reported that hydrophobic optics with decreased surface roughness and increased contact angle of acrylic IOLs reduce the number of adherent cells. Compared to PMMA IOLs, acrylic hydrophobic IOLs showed significantly less surface roughness and appear to be more suitable in preventing PCO (Chaudhury et al. 2010). Hydrophobic acrylic IOLs were reported to have more biocompatible surface characteristics than PMMA IOLs, and are favored by surgeons due to their outstanding performance in PCO prevention (Leaming 2004; Wilson et al. 2007). Furthermore, several studies have shown that modification of the IOL surface with a layer of heparin provides a more biocompatible surface with less cellular adhesion to the IOL than with an unmodified lens (Shan and Shalton 1995; Tanaka et al. 2000; Tognetto and Ravalico 2001). Recently, Abela-Formanek et al. (2011) investigated the biocompatibility of hydrophobic acrylic IOLs, silicone IOLs, and hydrophilic acrylic IOL with heparin surface modification in patients with uveitis who underwent cataract surgery. Higher uveal biocompatibility was achieved with the hydrophilic acrylic IOL with heparin surface modification compared to the hydrophobic acrylic IOL.

PCO is affected by the lens material. The material properties affect the biological response; IOL surface properties, such as the contact angle and roughness, are the most important factors influencing biocompatibility (Bertrand et al. 2014; Tanaka et al. 2005; Vasavada et al. 2011). Furthermore, biocompatibility, which is the response of living organisms to biomaterials, is dependent on the molecular interactions between the biomaterial surface and surrounding tissues. Therefore, an investigation of the chemical composition and molecular structure of IOLs for biocompatibility is necessary.

In the present study, we examine the physicochemical and surface properties of four currently available acrylic IOLs using static water contact angle, atomic force microscopy (AFM), Raman spectroscopy, and differential scanning calorimetry (DSC) measurements. This study is potentially relevant to surgeons faced with the task of choosing the most suitable IOL for a clinical or surgical situation. This information may assist IOL manufacturers in developing IOLs with the optimal characteristics.

## Materials and methods

### Sample preparation

**Fig. 2:**
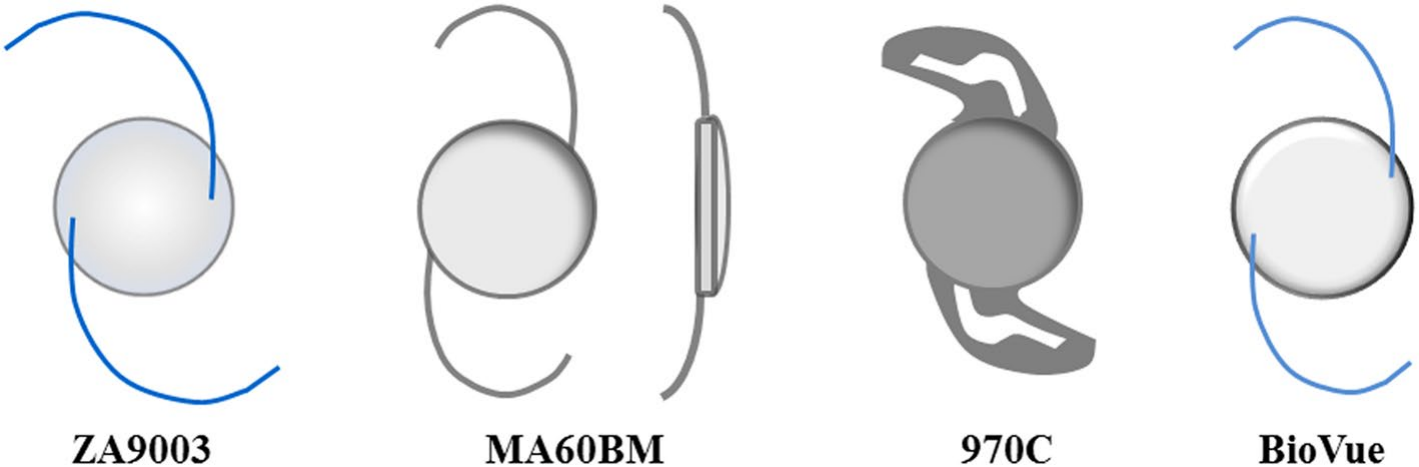
Four commercially available acrylic intraocular lenses (IOLs); ZA9003, MA60BM, 970C, and BioVue

Four commercially available acrylic IOLs including Tecnis^®^ three-piece hydrophobic acrylic IOL (ZA9003; AMO, Santa Ana, CA, USA), Acrysof^®^ three-piece hydrophobic acrylic IOL (MA60BM, Alcon Inc, Forth Worth, TX, USA), C-Flex^®^ hydrophilic acrylic IOL (970C, Rayner Inc, East Sussex, UK),and Ophthalmic Innovations International^®^ three-piece HSM hydrophilic acrylic IOL (BioVue, OII, Ontario, CA, USA), were used in the study (Table 1; Fig. 2). Before the measurement was performed, each IOL was dried. This study does not involve human or animal subjects.

**Table 1:** Intraocular lens (IOL) optics specifications

Manufacturer	Model	Diopter (D)	Material
AMO (Tecnis^®^)	ZA9003	22.5	Hydrophobic acrylic
ALCON (Acrysof^®^)	MA60BM	22.0	Hydrophobic acrylic
Rayner (C-Flex^®^)	970C	21.0	Hydrophilic acrylic
Ontario (OII^®^)	BioVue	22.0	HSM Hydrophilic acrylic

*OII* ^®^ Ophthalmic Innovations International^®^, *HSM* heparin-surface-modified

### Contact angle measurement

The static water contact angle was measured for information on the hydrophilicity of the four acrylic IOL samples. The contact angle measurements are based on the fact that the spreading of a drop on a surface is related to the physical–chemical forces between the liquid and the material (Dick et al. 2001). The wettability (i.e., hydrophilicity) is inversely proportional to the contact angle. The water contact angles of the IOLs were measured using a PHX150 contact angle analyzer (SEO Inc., Korea) in ambient humidity and temperature. First, approximately 1.0 L drop of deionized water was dropped onto the IOL surface. Second, digital images of the droplet were recorded and the contact angles were calculated from these images with software. The measurement was repeated 5 times for each IOL.

### AFM measurement

The surface roughness of the IOLs was examined using AFM (NANOS N8 NEOS, Bruker, Herzogenrath, Germany), which was operated in contact mode (nominal spring constant 0.2 N/m) in ambient air at room temperature in a liquid environment. All AFM images were scanned in 10 × 10 μm sections using a silicon cantilever with an integral pyramidal shaped tip (SICONG, Santa Clara, CA, USA). The different areas of the IOL surfaces were scanned at a resolution of 512 × 512 pixels and a scan speed of 1.0 line/s. To observe the surface roughness of the IOLs, the root mean square (RMS) surface roughness was calculated using Scanning Probe Image Processor (SPIP Version 4.8, Image Metrology, Denmark) software on 45 AFM topographic images for each group with a scan size of 10 × 10 μm.

### Raman spectroscopy

Raman spectroscopy (Ramanor T-64,000 microscopy system, JobinYvon, Longjumean, France) was performed to characterize the chemical composition and molecular structure of each IOL. An argon ion laser (514.5 nm) with a power of 50 mW was used as the excitation source. The spectra were recorded by scanning the 300–3400 cm^− 1^ region, which was accumulated for 10 scans with a 10 s acquisition time for each scan.

### Differential scanning calorimetry (DSC)

In general, the mechanical properties of most polymers, including acrylics, are affected by the temperature. It is important to provide the ideal temperature for optimal unfolding within the eye. The glass-transition temperature (T_g_) of the polymer networks with different compositions were determined using DSC (DSC Q2000, TA Instruments Inc., DE, USA) with a liquid nitrogen controller at a heating rate of 10 °C/min. All samples were run against an alumina reference in crimped aluminum pans with a temperature range of −40.0–150.0 °C.

### Statistical analysis

The results are expressed as the mean ± standard deviation (SD). One-way analysis of variance (ANOVA) was performed to compare the differences in the surface roughness of each IOL. Where appropriate, additional post-hoc comparisons were performed using a Student–Newman–Keuls test. A *P* value < 0.05 was considered significant.

## Results and discussion

### Wettability of the IOL surfaces

The contact angle measurement is a means to characterize surface properties and to correlate them to the biocompatibility of materials. The contact angle was measured to evaluate the hydrophilicity of the IOLs, as shown in Table 2. The contact angle (mean ± SD) showed similar values within each group. The hydrophobic acrylic IOLs, ZA9003 and MA60BM, had contact angles ranging from 77.9° ± 0.65° to 84.4° ± 0.09°. In the hydrophilic group, the contact angles ranged from 61.8° ± 0.45° (970C) to 69.7° ± 0.76° (BioVue). MA60BM had the highest value: 84.4° ± 0.09°. The HSM hydrophilic acrylic IOL (BioVue) showed a higher value than the non-HSM hydrophilic acrylic IOL, which may lead to a decrease in cellular adherence.

**Table 2:** Contact angle, surface roughness, and glass transition temperature of the four different intraocular lenses (IOLs)

Parameter	ZA9003	MA60BM	970C	BioVue	*p* value (ANOVA)
Contact angle (°)	77.9 ± 0.65^a^	84.4 ± 0.09^b^	61.8 ± 0.45^c^	69.7 ± 0.76^c^	<0.001
RMS roughness (nm)	7.53 ± 1.72^a^	11.80 ± 1.92^b^	14.14 ± 2.52^b^	5.87 ± 1.26^a^	<0.001
T_g_** (°C)	13.8	12.5	–	–	

Different superscript letters in the same parameter indicate significantly difference by ANOVA (Student–Newman–Keuls test, *p* < 0.05)

**T_g_ indicates glass transition temperature

The surface adhesiveness of a material to cells can be evaluated by measuring the surface contact angle, and the results showed that the hydrophobic optics of acrylic IOLs with a high contact angle may reduce the number of adherent cells (Abela-Formanek et al. 2002; Cunanan et al. 1998; Prinz et al. 2011). Furthermore, several studies have shown that fewer inflammatory cells and pigments are deposited on the HSM IOLs than on the non-HSM IOLs (Amon et al. 1996; Daynes et al. 2002; Fabrizius-Homan and Cooper 1991).

Contact angle measurements were performed to obtain information on the hydrophilicity of the lens materials, which can be used as a reliable parameter for the capsular biocompatibility. This information can assist in the choice of IOL based on the clinical situation.

### Surface roughness of IOL

The morphology and nanostructure of the IOL surfaces were analyzed using AFM. Detailed real-space topographical information on the surface features were provided in terms of the roughness values, which were defined as the RMS average of the height deviations taken from the mean data plane.

**Fig. 3:**
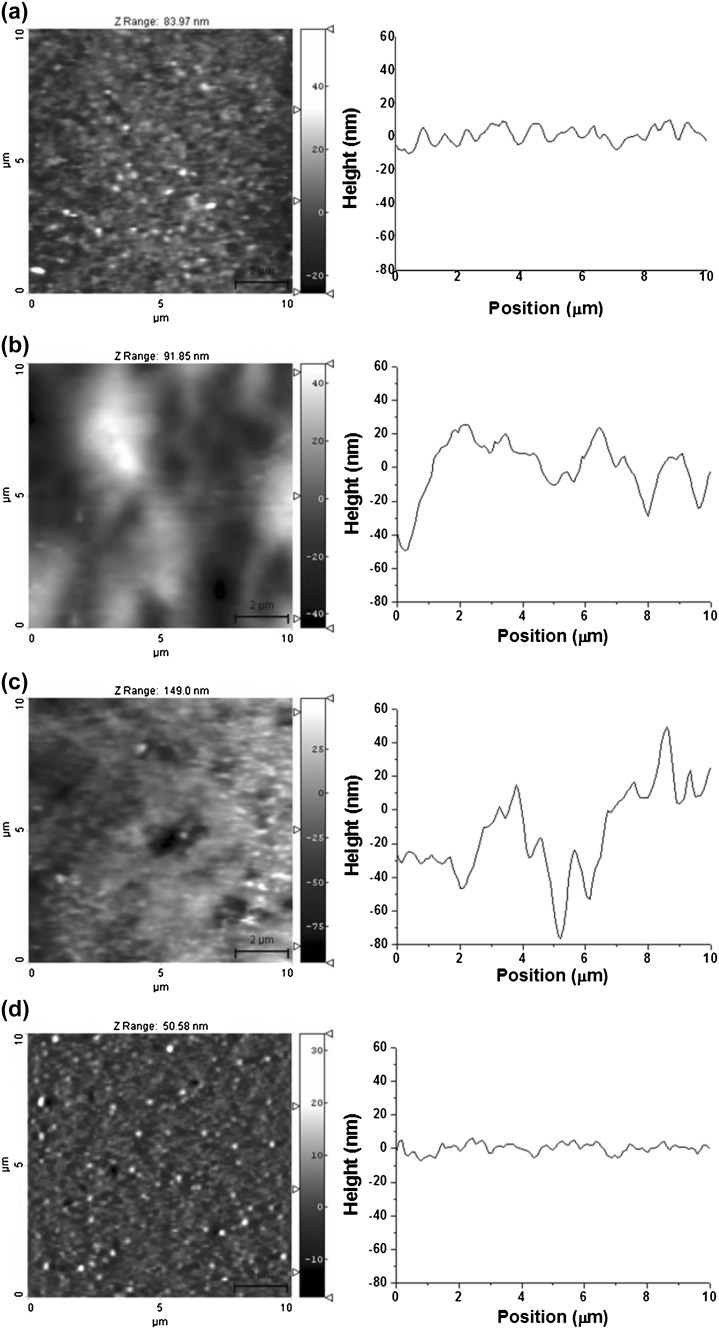
The atomic force microscopy (AFM) topography images and line profiles of the optic surfaces of **a** ZA9003 intraocular lens (IOL), **b** MA60BM IOL, **c** 970C IOL, and **d** BioVue IOL

Figure 3 shows representative AFM topographical images and line profiles of the four IOL surfaces. The surfaces of each IOL showed distinct nodule nanostructures with various depths and sizes due to the fabrication process. AFM is an effective and accurate tool for assessing the surface properties of IOLs on the nanometer scale. From the statistical results shown in Table 2, each IOL showed significant differences in surface roughness (*p* < 0.001, *n* = 45). The surface roughness of the HSM hydrophilic acrylic IOLs (BioVue) showed the lowest value of 5.87 ± 1.26 nm. This suggests that the BioVue IOL with HSM hydrophilic characteristics may lead to reduced cellular adhesion compared to the other IOL biomaterials because of its lower roughness value. Some studies reported that inflammatory cell adhesion to the IOL optical surface is affected by the roughness value of the IOL (Prinz et al. 2011; Yamakawa et al. 2003). IOLs with lower roughness values showed less cellular adhesion. In addition, considering that cell attachment and proliferation are decreased on negatively charged surfaces (Versural et al. 1991), HSM BioVue IOL may lead to reduced cellular adhesion due to the high negative charge density of heparin.

### Chemical properties of IOLs

Raman spectroscopy can provide details on the chemical composition and molecular structure of a biomaterial. Moreover, this technique has been utilized to monitor the effects of drugs on ocular diseases (Hosseini et al. 2003). The types of IOL can be identified from the unique peaks, intensities, and shapes in the Raman spectra.

**Fig. 4:**
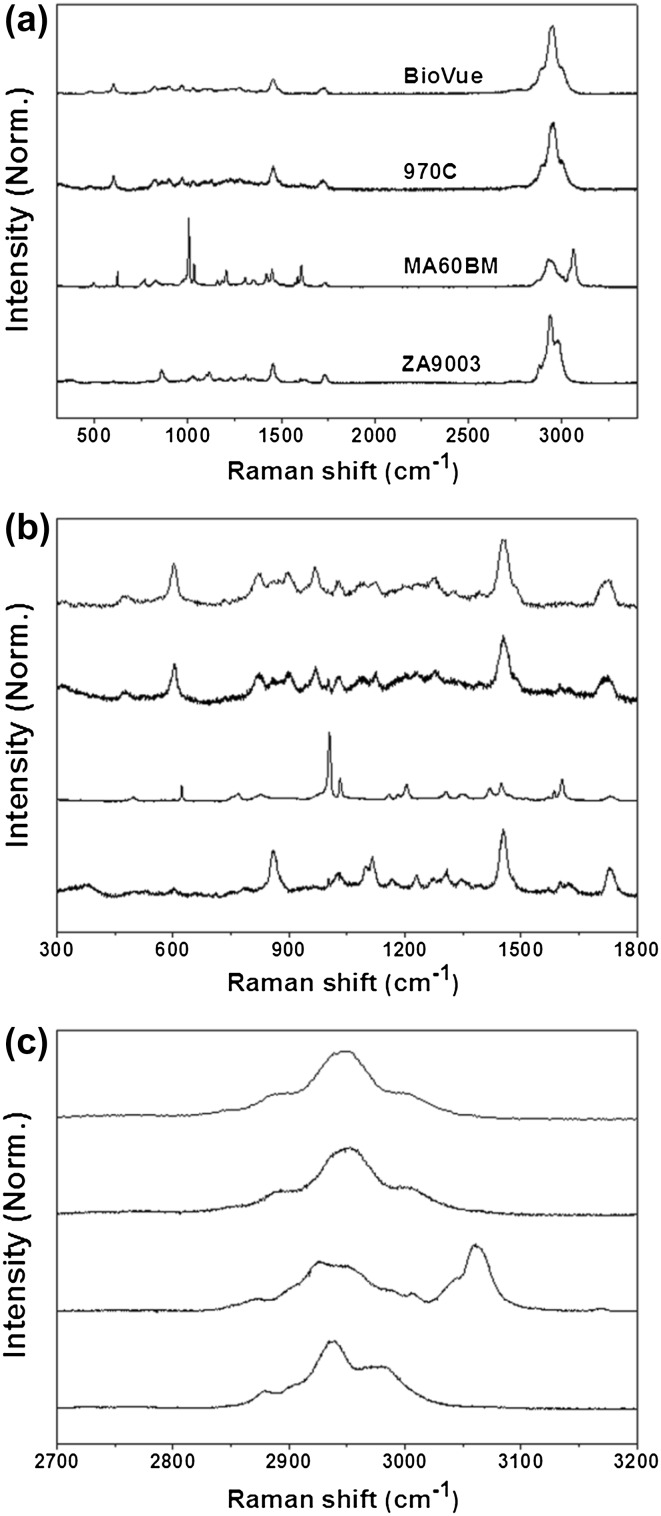
Raman spectral curves of intraocular lenses (IOLs) in three spectral ranges **a** 300–3400 cm^− 1^, **b** 300–1800 cm^− 1^, and **c** 2700–3200 cm^− 1^

Figure 4a shows the Raman spectra of all IOLs in the region, 300–3400 cm^− 1^. Figure 4b, c show the Raman spectra of the IOLs in the lower (300–1800 cm^− 1^) and higher (2700–3200 cm^− 1^) spectral range, respectively. Broad spectral peaks at approximately 1730 cm^− 1^ represents the C=O stretching mode of acrylates. The other fingerprint peaks in the range of approximately 860–1455 cm^− 1^ were similar to each other, even though the specific peak positions differed slightly. The fingerprint range from 1000 to 1400 cm^− 1^, which is mostly due to C–H bending modes, showed the distinctive feature of each IOL. The MA60BM IOL showed relatively high intensity at 1004 cm^− 1^, and had two peaks at 2930 and 3060 cm^− 1^ in the higher spectral range, corresponding to the C–H stretching and C=C–H stretching modes, respectively.

The Raman spectral characteristics of the hydrophilic acrylic IOL (970C) were similar to those of the HSM hydrophilic acrylic IOL (BioVue); they had peaks at 605, 1455, and 1730 cm^− 1^ in the lower spectral range and a relatively higher unique peak at 2950 cm^− 1^ in the higher spectral range. The hydrophilic acrylic IOLs showed distinctive peaks with different Raman shifts and peak heights compared to the hydrophobic acrylic IOLs. This suggests that although the basic material of each IOL is the same acrylate, each acrylic IOL has a different molecular structure because of the different fabrication process and different chemical composition of monomers and additives.

The chemical biocompatibility of the IOLs is responsible for the amount of residual monomer after the polymerization process. These monomers show dose dependent cytotoxicity, which is associated with the presence of a C=C band at 1640 cm^− 1^ due to the residual monomers (Galin et al. 1977). The Raman spectra of all IOLs showed the absence of a C=C band at 1640 cm^− 1^. This result suggests that all IOLs would be chemically biocompatible. Therefore, Raman spectroscopy would be a promising approach to the molecular characterization of biocompatibility, and be helpful in choosing the appropriate IOL material before surgery.

### Thermomechanical properties of IOLs

Glass transition temperature (T_g_) is the temperature at which an amorphous material passes from its rigid glassy state to its soft rubbery state (Moynihan et al. 1974). It is particularly important in the case of IOLs because it determines their ability to fold upon implantation in the eye. In general, the mechanical properties of acrylics and many other polymers are affected by temperature. Polymers show viscous flow at temperatures above T_g_, whereas they are hard and glassy below T_g_. Thus, lower the T_g_ of the material, more deformable and easily foldable the lens is (Bozukova et al. 2013). PMMA lenses have a T_g_ of approximately 110 °C, so this material has a brittle, glass-like characteristic at room temperature (Tehrani et al. 2004). The silicone lenses had T_g_ values of −91.7 and −119.6 °C, which indicates rubber-like characteristics at room temperature and very rapid unfolding within the eye (Tehrani et al. 2004).

In this study, DSC was used to examine the glass transition temperature of the various acrylic IOLs. As shown in Table 2, the two hydrophobic acrylic IOLs had similar T_g_ values: 13.8 °C for the ZA9003 IOL and 12.5 °C for the MA60BM IOL. This indicates that implantation at room temperature allows slower unfolding within the eye. In the two hydrophilic IOLs (970C and BioVue), distinct T_g_ values could not be obtained due to the broad DSC curves. Since the temperature of the operating room during cataract surgery is usually between 18 and 22 °C, IOLs with a T_g_ similar to the operating room temperature is required. The glass transition temperature correlates with the clinical intraoperative experience of unfolding within the eye, and helps determine the IOL material before surgery (Lee and Kim 2016; Tetz and Jorgensen 2015).

In summary, the physicochemical and surface properties of four common acrylic IOLs were investigated using contact angle, AFM, Raman spectroscopy, and DSC measurements. Each IOL type could be identified from their unique peaks, intensities, and shapes in the Raman spectra. The Raman spectra of all IOLs had no C=C band at 1640 cm^− 1^. This result suggests that all IOLs would be chemically biocompatible. The HSM hydrophilic acrylic IOLs (BioVue) showed the lowest surface roughness value, which may lead to lower cellular adhesion compared to the other IOLs. Furthermore, since heparin has highest negative charge density of any known biological molecule, the BioVue IOL may lead to a decrease in cell attachment and proliferation. Cell adhesion properties of the tested IOLs will be assessed in future studies.

Recently, there has been interest in the use of IOLs as drug reservoirs, which appears to be a promising way to treat inflammation, infection, and posterior capsule opacification after cataract surgery (Davis et al. 2012; Liu et al. 2013; Matsushima et al. 2005). Since an IOL is implanted during cataract surgery and remains in the eye after surgery, it is an ideal delivery system for intraocular lens containing a drug. The drug can be loaded on the IOL via presoaking/coating or attaching the drug reservoir onto the IOL haptic or optic. Kleinmann et al. evaluated the ability and safety of a hydrophilic acrylic IOL as a drug delivery system for commercially available gatifloxacin and moxifloxacin using the presoaked method (Kleinmann et al. 2006). They reported that no eye showed signs of clinical toxicity. Results showed the IOL is a safe and effective drug-delivery system.

Therefore, the study of IOL materials is very important for further industrial manufacture of reliable biocompatible IOLs. Furthermore, these results could help in choosing the most suitable IOL for a clinical or surgical situation.

## Conclusion

In this study, the physicochemical and surface properties of four currently available acrylic IOLs were investigated by using static water contact angle, AFM, Raman spectroscopy, and DSC measurements. Based on the Raman spectra of IOLs, all IOLs would be chemically biocompatible. The hydrophobic acrylic IOLs (ZA9003 and MA60BM) exhibited glass transition temperatures at around room temperature, which indicates appropriate temperature for the optimal unfolding in cataract surgery. Among the tested IOLs, BioVue that is the hydrophilic acrylic foldable lens with heparin surface modification showed the lowest surface roughness value. This result may lead to lower cellular adhesion compared to the other IOLs, and would be a suitable for a clinical application.
